# Automated detection of poor-quality data: case studies in healthcare

**DOI:** 10.1038/s41598-021-97341-0

**Published:** 2021-09-09

**Authors:** M. A. Dakka, T. V. Nguyen, J. M. M. Hall, S. M. Diakiw, M. VerMilyea, R. Linke, M. Perugini, D. Perugini

**Affiliations:** 1 Presagen, Adelaide, SA 5000 Australia; 2 School of Mathematical Sciences, The University of Adelaide, Adelaide, SA 5000 Australia; 3 School of Computing and Information Technology, University of Wollongong, Wollongong, NSW 2522 Australia; 4 Australian Research Council Centre of Excellence for Nanoscale BioPhotonics, Adelaide, SA 5000 Australia; 5 Ovation Fertility, Austin, TX 78731 USA; 6 Texas Fertility Center, Austin, TX 78731 USA; 7 Department of Medical Imaging-SAMI, Women’s and Children’s Hospital Campus, Adelaide, SA 5000 Australia; 8 Adelaide Medical School, The University of Adelaide, Adelaide, SA 5000 Australia

**Keywords:** Medical imaging, Mathematics and computing

## Abstract

The detection and removal of poor-quality data in a training set is crucial to achieve high-performing AI models. In healthcare, data can be inherently poor-quality due to uncertainty or subjectivity, but as is often the case, the requirement for data privacy restricts AI practitioners from accessing raw training data, meaning manual visual verification of private patient data is not possible. Here we describe a novel method for automated identification of poor-quality data, called Untrainable Data Cleansing. This method is shown to have numerous benefits including protection of private patient data; improvement in AI generalizability; reduction in time, cost, and data needed for training; all while offering a truer reporting of AI performance itself. Additionally, results show that Untrainable Data Cleansing could be useful as a triage tool to identify difficult clinical cases that may warrant in-depth evaluation or additional testing to support a diagnosis.

Advances in deep learning using artificial neural networks (ANN)^1,2^ have resulted in the increased use of AI for healthcare applications^3–5^. One of the most successful examples of deep learning has been the application of convolutional neural network (CNN) algorithms for medical image analysis to support clinical assessment^6^. AI models are trained with labeled or annotated data (medical images) and learn complex features of the images that relate to a clinical outcome, which can then be applied to classify new unseen medical images. Applications of this technology in healthcare span a wide range of domains including but not limited to dermatology^7,8^, radiology^9,10^, ophthalmology^11–13^, pathology^14–16^, and embryo quality assessment in IVF^17^.

Despite the enormous potential of AI to improve healthcare outcomes, AI performance can often be sub-optimal as it is crucially dependent on the quality of the data. While AI practitioners often focus on the quantity of data as the driver of performance, even fractional amounts of poor-quality data can substantially hamper AI performance. Good-quality data is therefore needed to train models that are both accurate and generalizable, and which can be relied upon by clinics and patients globally. Furthermore, because measuring AI performance on poor-quality test data can mislead or obfuscate the true performance of the AI, good-quality data is also important for benchmark test sets used in performance reporting, which clinics and patients rely on for clinical decisioning.

We define two types of poor-quality data:

- **Incorrect data**: Mislabeled data, for example an image of a dog incorrectly labeled as a cat. This also includes adversarial attacks by intentionally inserting errors in data labels (especially detrimental to online machine learning methods^18^).
- **Noisy data**: Data itself is of poor quality (e.g. out-of-focus image), making it ambiguous or uninformative, with insufficient information or distinguishing features to correlate with any label.

In healthcare, clinical data can be inherently poor quality due to subjectivity and clinical uncertainty. An example of this is pneumonia detection from chest X-ray images. The labeling of a portion of the image can be somewhat subjective in terms of clinical assessment, often without a known ground truth outcome, and is highly dependent on the quality of the X-ray image taken. In some cases, the ground truth outcome might also involve clinical data that is not present in the dataset used for analysis, such as undiagnosed conditions in a patient, or effects that cannot be seen from images and records provided for the assessment. This kind of clinical uncertainty can contribute to both the incorrect and noisy data categories. Therefore, poor-quality data cannot always be reliably detected, even by clinical experts. Furthermore, due to data privacy, manual visual verification of private patient data is not always possible.

Several methods exist to account for these sources of reader variability and bias. One method^19^ uses a so-called Direct Uncertainty Prediction to provide an unbiased estimate of label uncertainty for medical images, which can be used to draw attention to images requiring a second medical opinion. This technique relies on training a model to identify cases with high potential for expert disagreement. Other methods model uncertainty in poor-quality data through Bayesian techniques^20^. However, such techniques require significant amounts of annotated data from multiple experts, which is often not readily available. Some methods assume that erroneous label distribution is conditionally independent of the data instance given the true label^21^, which is an assumption that does not hold true in the settings considered in this article. Other techniques^22^ relax this assumption by using domain-adapted generative models to explain the process that generates poor-quality data, though these techniques typically require additional clean data to generate good priors for learning. This is an issue in medical domains such as the assessment of embryo viability, where reader variability is significant^17^ and ground truth labels may be impossible to ascertain, so there is no way of identifying data as poor quality a priori. There is a need for better approaches to cleanse poor-quality data automatically and effectively, in and beyond healthcare.

## Results

In this paper, a novel technique is presented for automated data cleansing which can identify poor data quality without requiring a cleansed dataset with known ground truth labels. The technique is called Untrainable Data Cleansing (UDC) and is described in the Methods section. UDC essentially identifies and removes a subset of the data (i.e. cleanses the data) that AI models are unable to correctly label (classify) during the AI training process. From a machine learning perspective, the two types of poor-quality data described above are realized through: (1) identifying data that are highly correlated to the opposite label of what would reasonably be expected, based on the classification of most of the data in the dataset (incorrect data); or (2) identifying data that have no distinguishing features that correlate with any label (noisy data). Results show that UDC can consistently and accurately identify poor-quality data, and that removal of UDC-identified poor-quality data, and thus “cleansing” of the data, ultimately leads to higher performing and more reliable AI models for healthcare applications.

### Validation of UDC

UDC was first validated using two types of datasets, cats and dogs for binary classification, and vehicles for multi-classification. These datasets were used because the ground truth can be manually confirmed, and therefore incorrect labels could be synthetically injected.

#### Binary classification using cats and dogs

A benchmark (Kaggle) dataset of 37,500 cat and dog images were used to validate UDC for binary classification. This dataset was chosen because the ground truth outcomes (labels) could be manually determined with certainty, and synthetic incorrect labels could be readily introduced by flipping the correct label to an incorrect label. Synthetic incorrect labels were added to this dataset to test UDC under various amounts of poor-quality data.

A total of 24,916 images (12,453 cats, 12,463 dogs) were used for training and 12,349 images (6143 cats, 6206 dogs) used as a separate blind test set. Synthetic errors (incorrect labels) were added to the training dataset (but not the test set), which was split 80/20 into training and validation sets. A single round of UDC was applied to the training dataset, poor-quality data identified by UDC was removed, and a new AI model was trained on the UDC-cleansed dataset. The highest balanced AI accuracy achievable on the blind test dataset was reported. Results are shown in Table 1.

**Table 1:** Results from a single round of UDC applied to various incorrect label levels.

Incorrect label levels (cats %, dogs %)	(0%, 0%)	(35%, 5%)	(50%, 5%)	(30%, 30%)
Average level of incorrect labels (%)	0.0	20.0	27.5	30.0
Original balanced accuracy (%)	99.2	77.9	72.6	63.1
UDC balanced accuracy (%)	–	98.3	98.7	94.7
Accuracy improvement (%)	–	**20.4**	**26.1**	**31.6**
Images removed by UDC (Total)	–	4699	6757	7578
Images removed by UDC (%)	–	18.9	27.1	30.4

The bold font is to draw attention of the reader that there is an accuracy improvement.

Cats % is the percentage of cat images incorrectly labeled as dogs, vice versa. Baseline model is assumed to contain 0% error in both classes, denoted (0%, 0%).

Results show that UDC is resilient to even extreme levels of errors, functioning in datasets with up to 50% incorrect labels in one class (while the other class remained relatively clean), and with significant symmetric errors of up to 30% incorrect labels in both classes. Visual assessment verified removal of both incorrect data and a minor proportion of noisy data, where, for example, dogs looked like cats (see Supplementary Figure S1 online). Improvements of greater than 20% were achieved in all cases. Compared to cases with uniform levels of incorrect labels, those with asymmetric incorrect labels, e.g. (35%, 5%), achieved a higher balanced accuracy of over 98% after just one round of UDC. This is expected, since in the asymmetric cases, one class remains as a true correct class, allowing UDC to be more confident in identifying incorrectly labeled samples.

In the uniform cases a slightly lower balanced accuracy of 94.7% was achieved after one round of UDC, which was found to identify and remove 88% of the intentionally mislabeled images. A second round of UDC improved upon the results of a single application of UDC, successively increasing the predictive power of the dataset by removing the remaining incorrect data. The accuracy achieved after a second round of UDC (99.7%) to the symmetric case (30%, 30%) showed an improvement even when compared to the baseline accuracy (99.2%) on datasets with 0% synthetic error. Further tests would be required to confirm the statistical significance of this uplift, but it is not unreasonable that the UDC could filter out noisy data that may be present in the original clean dataset (since the baseline set itself is not guaranteed to be free of poor-quality data), therefore helping to not only recover but surpass the accuracy of models trained on the baseline datasets.

For the symmetrical (50%, 50%) case (not shown), UDC simply learns to treat one entire class as incorrect and the other as correct, thereby discarding all samples from the opposite class as data errors. Therefore, as might be expected, UDC fails when data error levels in both classes approach 50%, because there is no longer a majority of good-quality data to allow UDC to confidently identify the minority of poor-quality data. In this case, the dataset is deemed unsuitable for AI training. To address such datasets that are so noisy as to have virtually no learnable features, an important screening process prior to implementing a UDC workflow, is to conduct a hyperparameter search to determine parameter spaces wherein predictive power can be achieved on a given dataset. The hyperparameter search is implemented by selecting a range of architectures, learning rates, momentum values and optimizers, and measuring the accuracy of each model on a validation set, at each epoch while training, for a range of 200–300 epochs. Hyperparameter combinations are eligible for inclusion in the UDC workflow if their associated training runs include a high mean accuracy value across the epochs for training, and are screened if they are not able to achieve a statistically significant accuracy above 50%.

#### Multi-classification using vehicles

An open image database of 27,363 vehicles was used to validate UDC for multi-classification. The dataset comprised four classes of vehicles: airplanes, boats, motorcycles, and trucks. A total of 18,991 images (7244 airplanes, 5018 boats, 3107 motorcycles, 3622 trucks) were used for training, and 8372 images (3101 airplanes, 2194 boats, 1424 motorcycles, 1653 trucks) used as a separate blind test set. As in the previous section, this dataset was chosen because the ground truth outcomes (labels) could be manually ascertained, and synthetic incorrect labels could be readily introduced. Synthetic incorrect labels were uniformly added to each class in the training dataset in increments of 10% to test UDC under various amounts of poor-quality data. UDC results are shown in Supplementary Figure S2 online. This figure shows, for a given data error rate (0–90%), the number of images that are correctly predicted by *x* constituent UDC models, where the *x*-axis ranges from 0 to the total number of models used in the UDC workflow. Each UDC histogram is thus quantized by the total number of models, and each bin shows a different total number of images, depending on the choice of data error rate.

Results are summarized in Table 2, which shows the percentage improvement for all cases after only a single round of UDC, removing both noisy and incorrect labels. Errors are calculated as the standard error on the mean for results obtained from eight models overall to reduce bias on a particular validation set (four model architectures based on Residual Convolutional Neural Network (ResNet)^23^ and Dense Convolutional Network (DenseNet)^24^, each trained on two cross-validation phases).

**Table 2:** Results from a single round of UDC applied to various incorrect label levels.

Error rate (%)	Before UDC accuracy (%)	After UDC accuracy (%)	Enhancement (%)
0	**98.8** ± **0.1**	**98.8** ± **0.1**	0.0 ± 0.1
10	97.5 ± 0.2	**98.6** ± **0.1**	**1.1** ± **0.2**
20	96.7 ± 0.3	**98.5** ± **0.1**	**1.8** ± **0.3**
30	94.7 ± 0.8	**98.2** ± **0.1**	**3.5** ± **0.8**
40	92.8 ± 0.5	**98.3** ± **0.1**	**5.5** ± **0.5**
50	87.7 ± 1.9	**98.1** ± **0.1**	**10.3** ± **1.9**
60	74.7 ± 2.6	**97.0** ± **0.2**	**22.3** ± **2.6**
70	47.4 ± 2.5	**92.2** ± **0.6**	**44.8** ± **2.6**
80	**15.3** ± **2.0**	3.7 ± 0.5	-11.7 ± 2.0
90	**2.9** ± **0.6**	1.2 ± 0.2	-1.7 ± 0.7

The bold font draws attention to whether the 'Before UDC' value or the 'After UDC' value is the greater, for each scenario, so that it is easy for the reader to judge which rows there was an improvement, and which rows there was a decrease.

Incorrect labels are distributed evenly across all four classes: airplanes, boats, motorcycles, and trucks. UDC is robust to label error rates up to 70%, where a 44.8% gain in AI performance is achieved after a single round of UDC.

For the multi-class case, results show that UDC is resilient and can identify poor-quality data and improve AI accuracy even at more extreme levels of errors (i.e. 30–70%) compared with the binary case. UDC fails when the percentage of incorrect labels in each class approaches 80%. This is because when 80% or more of a class’ labels are distributed into three other classes, this results in fewer correct labels than incorrect labels for that class, making model training impossible as the model is pulled away from convergence by a larger number of incorrectly vs. correctly labeled data, and making such data *uncleansable*.

Taken together, these results suggest that UDC creates cleansed datasets that can be used to develop high performing AI models that are both accurate and generalizable using fewer training data, and reduced training time and cost. Near baseline-level performance was achieved using datasets containing up to 70% fewer training data. We showed that 97% accuracy could be achieved on datasets with up to 60% fractional incorrect labels for all classes, using less than 30% of the original amount of training data. In an even more extreme case with 70% incorrect labels, UDC had a higher false positive rate (correct labels images identified as incorrect), which resulted in the removal of 95% of the original dataset, but models trained on the remaining 5% still achieved over 92% accuracy on a blind test set. Finally, application of UDC gave greater stability and accuracy during the training process (across epochs), which means that AI model selection for deployment can be automated because the selection is not hyper-dependent on a training epoch once a threshold of accuracy is achieved.

### Application of UDC

The UDC technique was then applied to two healthcare problems, pediatric chest X-ray images for identification of pneumonia, and embryo images to identify likelihood of pregnancy (viability) for in vitro fertilization (IVF). Finally, UDC was also shown to be able to cleanse benchmark test datasets themselves to enable a truer and more realistic representation of AI performance.

#### Assessment of pediatric chest X-rays for pneumonia detection

A publicly available dataset of pediatric chest X-ray images with associated labels of “Pneumonia” or “Normal” from Kaggle^25^ was used. The labels were determined by multiple expert physicians. There were 5232 images in the training set and 624 images in the test set. UDC was applied to all 5856 images. Approximately 200 images were identified as noisy, while no labels were identified as incorrect. This suggests there were no suspected labeling errors in the dataset, but the images identified by UDC were considered poor-quality or uninformative. Poor-quality images in this dataset mean that labels of “normal” or “pneumonia” were not easily identifiable with certainty from the X-ray.

**Figure 1:**
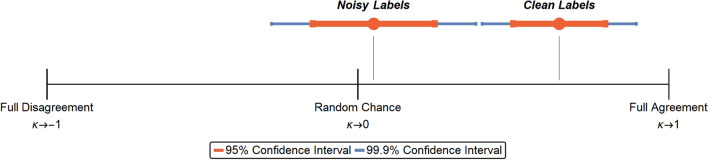
Cohen’s kappa test for noisy and Correct labels shows that images with Correct labels lead to a significantly higher level of agreement than random chance, and significantly higher than those with noisy labels.

To verify the result, an independent expert radiologist assessed 200 X-ray images from this dataset, including 100 that were identified as noisy by UDC, and 100 that were identified as correct. The radiologist was only provided the image, and not the image label nor the UDC assessment. Images were assessed in random order, and the radiologist’s assessment of the label for each image recorded. Results showed that the reader consensus between the radiologist’s label and the original label was significantly higher for the correct images compared with the noisy images. Applying Cohen’s kappa test^26^ on the results gives levels of agreement for noisy ($\kappa \approx 0.05$) and correct ($\kappa \approx 0.65$) labels (refer to Fig. 1). This confirms that for noisy images detected by UDC, there is insufficient information in the image alone to conclusively (or easily) make an assessment of pneumonia by either the radiologist or the AI. UDC could therefore prove beneficial as a screening tool for radiologists that could help triage difficult to read or suspicious (noisy) images that warrant further in-depth evaluation or additional tests to support a definitive diagnosis.

**Figure 2:**
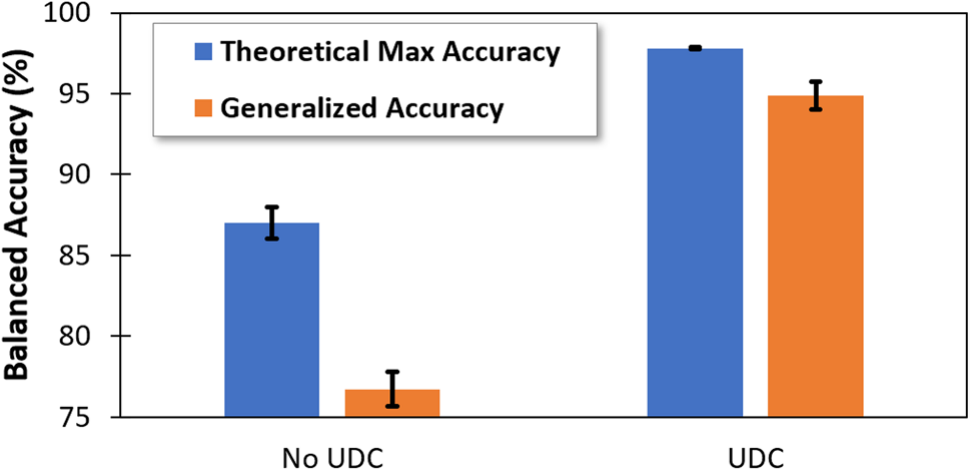
Balanced accuracy before and after UDC. The orange bar represents the AI accuracy on the test dataset using the standard AI training practice. The blue bar represents the theoretical maximum AI accuracy possible on the test dataset. The discrepancy between these two values is indicative of the generalizability of the model.

We then compared AI performance when trained using the original uncleansed X-ray training dataset versus UDC-cleansed X-ray training dataset with noisy images removed. Results are shown in Fig. 2. The blue bar in the figure represents a theoretical maximum accuracy possible on the test dataset. It is obtained by testing every trained AI model on the test dataset to find the maximum accuracy that can be achieved. The orange bar is the actual (generalized) accuracy of the AI obtained using standard practice for training and selecting a final AI model using the validation set, then testing AI performance on the test set. The difference between the blue bar and orange bar indicates the generalizability of the AI, i.e. the ability of the AI to reliably apply to unseen data. Results show that training the AI on a UDC-cleansed dataset increases both the accuracy and generalizability of the AI. Additionally, the AI trained using a UDC-cleansed dataset achieved 95% generalized accuracy. This exceeds the 92% accuracy reported for other models in the literature using this same chest X-ray dataset^27^.

**Figure 3:**
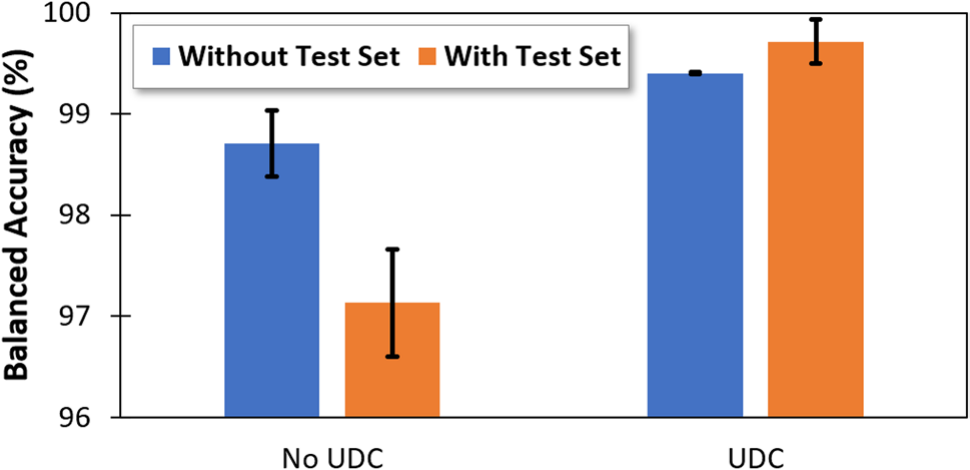
The colors of the bars represent the performance of the model on the validation set, with (orange) and without (blue) the test set included in the training set. AI performance drops when the uncleansed blind test set is included in the training set, indicating a considerable level of poor-quality data in the test set.

Lastly, we investigated application of UDC on the test dataset of X-ray images to assess its quality. This is vital because the test dataset is used by AI practitioners to assess and report on AI performance. Too much poor-quality data in a test set means the AI accuracy is not a true representation of AI performance. To evaluate this, we injected the uncleansed test dataset into the training dataset used to train the AI to determine the maximum accuracy that could be obtained on the validation dataset. Figure 3 shows reduced performance of AI trained using the aggregated dataset (training dataset plus the noisy test dataset) compared with the AI trained only using the cleansed training set. This suggests that the level of poor-quality data in the test dataset is significant, and thus care should be taken when AI performance is measured using this particular test dataset.

**Figure 4:**
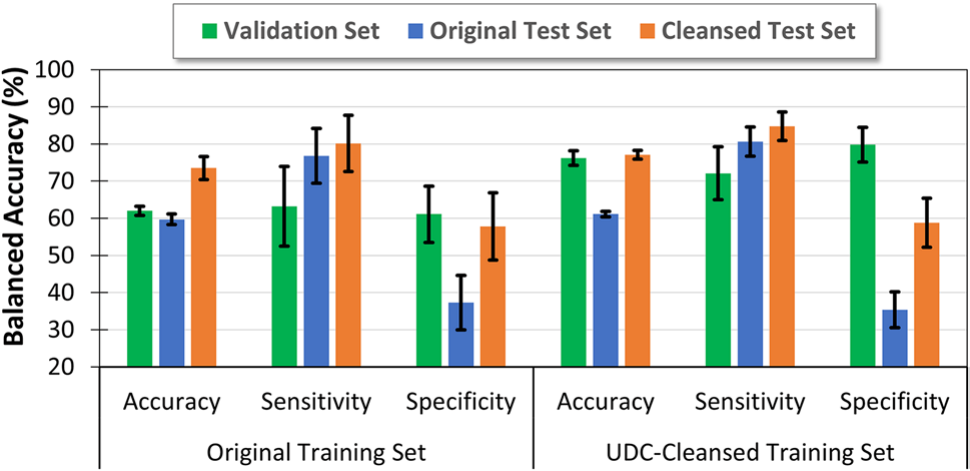
Performance metrics of AI model predicting clinical pregnancy, trained on original (left section) and UDC-cleansed (right section) training data. Both graphs show results on the validation set (green), and corresponding original test set (blue) and UDC-cleansed test set (orange).

#### Assessment of embryo quality for IVF

Finally, UDC was successfully applied to the problem of assessing embryo viability in IVF. UDC was a core technique in developing a commercial AI healthcare product, which is currently being used in IVF clinics globally^17^. The AI model analyzes images of embryos at Day 5 of development to identify which ones are viable and likely to lead to a clinical pregnancy.

Clinical pregnancy is measured by the presence of a fetal heartbeat at the first ultrasound scan approximately 6 weeks after the embryo is transferred to an IVF patient. An embryo is labeled viable if it led to pregnancy, and non-viable if a pregnancy did not occur. Although there is certainty in the outcome (pregnancy or no pregnancy), there is uncertainty in the labels, because there may be patient medical conditions or other factors beyond embryo quality that prevent pregnancy (e.g. endometriosis)^17^. Therefore, an embryo that is viable may be incorrectly labeled as non-viable. These incorrect labels impact the performance of the AI if not identified and removed.

UDC was applied to images of embryos to identify incorrect labels. These were predominantly in the training dataset’s non-viable class, as expected, as they included embryos that appeared viable but were labeled as unviable. Performance results are shown in Fig. 4. AI models trained using a UDC-cleansed training dataset achieved an increase in accuracy, from 59.7 to 61.1%, when reported on the standard uncleansed test dataset. This small increase in accuracy was not statistically significant, but could potentially be misleading, as the uncleansed test set itself may comprise a significant portion of incorrectly labeled non-viable embryo images, thus reducing specificity as the AI model improves. For the predominantly clean viable class, sensitivity increased by a larger amount, from 76.8 to 80.6%. When a UDC-cleansed test set is utilized, AI models trained using a UDC-cleansed training dataset achieved an increase in accuracy from 73.5 to 77.1%. This larger increase is a truer representation of the AI performance, and while the uplift is just at the $1-\sigma$ level, it is noted that a medical dataset may require multiple rounds of UDC to fully cleanse the training set.

Effect sizes before and after UDC are represented using Cohen’s *d*, as shown in Table 3, along with *p*-values. Effect sizes larger than 0.6 are considered “large”, meaning that for all test sets (including validation), UDC has a large effect on training and inference (test) performance, except for specificity results for both cleansed and uncleansed (expected due to the large proportion of incorrectly labeled non-viable embryos) test sets. This can be interpreted as there being a significant pair-wise uplift in sensitivity without much cost to specificity. In all cases, there is very large ($d>1.4$) effect on overall performance. Taken together these results suggest that using UDC to cleanse training datasets can improve the accuracy of the AI even in clinical datasets with a high level of mislabeled, poor-quality data.

**Table 3:** Effect size *d* and *p*-value of enhancement after application of UDC on embryo dataset.

Dataset	Validation set			Original test set			Cleansed test set		
Test statistic	Acc.	Sens.	Spec.	Acc.	Sens.	Spec.	Acc.	Sens.	Spec.
*d*	8.656	2.439	3.234	1.414	0.664	− 0.329	1.611	0.788	0.144
*p*	< 0.001	0.199	0.049	0.002	0.401	0.449	0.062	0.385	0.482

## Discussion

This study characterizes a novel technique, Untrainable Data Cleansing (UDC), that serves to automatically identify, and thus allow removal of, poor-quality data to improve AI performance and reporting. In the clinical setting, accurate AI could mean the difference between life and death, or early diagnosis versus missed diagnosis. Thus it is critical that poor-quality data are identified and removed so as not to confuse the AI training process and impact clinical outcomes. It can be difficult for even the most experienced clinicians to identify poor-quality data, particularly when the clinical outcome is uncertain, or the quality and integrity of the data does not allow for a definitive labeling of the image. Furthermore, due to data privacy laws, it may not even be possible to manually assess data quality of private medical datasets. Because UDC can be “shipped” to the secure location of private data, it offers an automated way of addressing data quality concerns while respecting data privacy laws.

UDC was validated across two problem sets, (1) cats vs. dogs, and (2) vehicles, or binary and multi-classification problems, respectively, because image labels could be manually verified. In both cases UDC was effective at identifying synthetically introduced incorrect labels. Training AI models following removal of poor-quality data significantly improved the AI performance, in terms of both accuracy and generalizability. UDC was applied to two medical problem sets, one for pneumonia detection in chest X-rays, and the other for embryo selection in IVF. Both are challenging clinical assessment areas due to varying degrees of noisy or incorrectly labeled data. In both studies UDC was effective (as measured on double blind datasets) at identifying poor quality data, and yielded significant improvements in accuracy and generalizability.

In UDC, the use of a variety of model architectures as well as *k*-fold cross-validation serves to mitigate overfitting on smaller datasets. Though there may always be a trivial lower bound on dataset size, the behavior of UDC as total training images decreases was found to be stable after training on (cleansed) datasets as low as 5% the size of the initial training datasets. Nevertheless, to further alleviate the effect of overfitting, all models are trained using data augmentation, dropout, weight balancing, learning rate scheduling, weight decay, and early stopping.

In the same way that poor-quality training data can impact AI training, the reporting (or testing) of AI performance done so on a poor-quality test dataset (that contains noisy or incorrectly labeled data) can lead to inaccurate performance reporting. Inaccurate reporting can mislead clinicians and patients on the true performance and reliability of the AI, with potential real-world consequences for those that may rely on AI results. We assessed the utility of UDC for cleansing test datasets and showed that the accuracy of the AI reported on test datasets cleansed with UDC was different to that reported on uncleansed test datasets. The reporting of AI accuracy on a UDC-cleansed test set was shown to be a truer representation of the AI performance based on independent assessment.

Since UDC relies on pooling or ensembling various model architectures, its advantages (namely, that it is relatively agnostic to initial feature-crafting and allows for automated implementation) could be enhanced with the application of discriminant analysis techniques to provide richer context when identifying noisy or incorrect data. Future research is possible investigating how the granularity of Principal Component Analysis-Linear Discriminant Analysis (PCA-LDA) or related techniques, which cannot be applied directly to images themselves, but to model activation (or feature) layers, could be applied to more precisely explain why individual samples were identified as poor-quality by UDC. Such methods may be able to identify which features in each image are not in agreement with the received label.

Finally, we showed that UDC was able to identify noisy data, which in the case of the pneumonia X-rays neither the AI nor the radiologist could consistently classify. The ability for UDC to identify these cases suggests it can be used as a triage tool and direct clinicians to those cases that warrant new tests or additional in-depth clinical assessment. This study demonstrates that the performance of AI for clinical applications is highly dependent on the quality of clinical data, and the utility of a method like UDC that can automatically cleanse otherwise poor-quality clinical data cannot be overstated.

## Methods

### UDC algorithm

Untrainable Data Cleansing (UDC) can identify three categories of image-label pairs:

- **Correct**—strongly expected to be correct (i.e. label matches the ground-truth).
- **Incorrect**—strongly expected to be incorrect (i.e. label does not match ground-truth).
- **Noisy**—data is ambiguous or uninformative to classify with certainty (i.e. label may or may not match ground-truth).

UDC delineates between correct, incorrect, and noisy images using the method described in Algorithm 1, which utilizes a mixture between sampling different (n) model architectures and sampling across data using *k*-fold cross validation (KFXV). These $n\times k$ models vote on each image-label in this manner to reduce bias and to increase robustness to outliers.
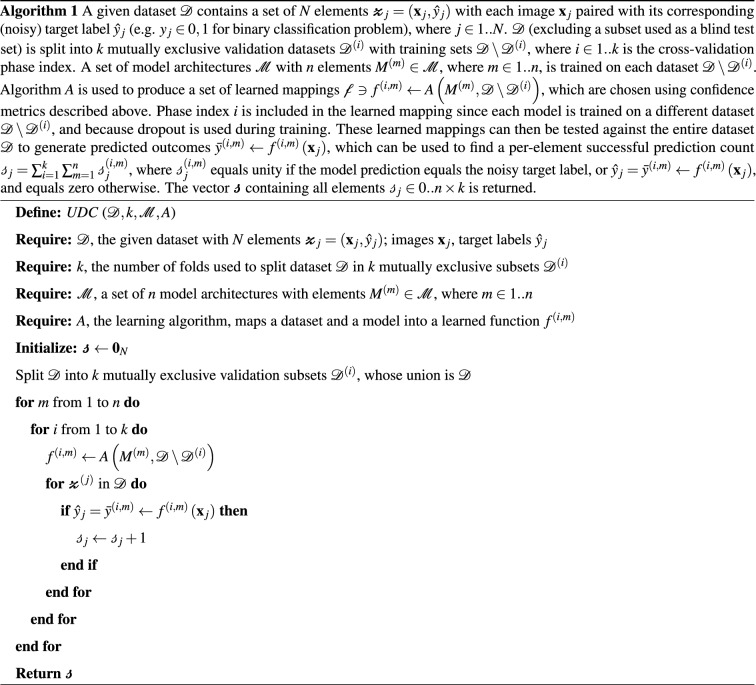


Prior to training each AI model configuration, the images are minimally pre-processed by auto-scaling their resolutions to $224 \times 224$ pixels, and normalizing the color levels using the standard ImageNet mean RGB values of (0.485, 0.456, 0.406) and standard deviations of (0.229, 0.224, 0.225), respectively.

We describe UDC as “*turning AI onto itself*”, as it uses the AI training process to identify poor-quality data. Multiple AI models using different architectures and parameters are trained using the data (to be cleansed), then the AI models are applied back on the same training data to infer their labels. Data which cannot be consistently classified correctly are identified as poor-quality (i.e. incorrect or noisy).

The idea behind UDC is that if data cannot be consistently correctly classified within the AI training process itself, which is where AI models are likely to find the strongest correlations and correct classifications on the dataset used to train/create it, then the data is likely to be poor-quality.

The intuition behind using UDC to subdivide image-label pairs into three types of labels is based on probability theory. A correct label can be thought of as a positively weighted coin ($p\gg 0.5$), where p is the probability of being correctly predicted by a certain model. In contrast, an incorrect label can be thought of as a negatively weighted coin ($p\ll 0.5$)—very likely to be incorrectly predicted. A noisy label can be thought of as a fair coin ($p\approx 0.5$)—equally likely to be correctly or incorrectly predicted. To illustrate how this intuition applies to UDC, we consider a hypothetical dataset of *N* image-label pairs. Algorithm 1 is applied to this dataset to produce a number of successful predictions ($s_j$) for each image *j*. A histogram of $s_j$ values (with increasing *s* on the *x*-axis) then shows how correct, incorrect, and noisy labels tend to cluster at high, low, and medium values of *s*, respectively.

Synthetic errors (incorrect labels) were added to the training dataset of 24,916 images, which is split 80/20 into training and validation sets with the following parameters used for each study *t*, where ${\varvec{\mathscr {n}}}^{(t)}=\left( {\varvec{\mathscr {n}}}_{cat}^{(t)},{\varvec{\mathscr {n}}}_{dog}^{(t)}\right)$, where $0\le {\varvec{\mathscr {n}}}(\%) \le 100$, contains the fractional level of incorrect labels for cat and dog classes (see Table 4), respectively, and where RN stands for ResNet^23^, and DN stands for DenseNet^24^.

**Table 4:** Parameters used to test untrainable data cleansing (algorithm 1) on cats and dogs dataset.

*k*	*n*	${{\mathcal {M}}}^{(1)}$	${{\mathcal {M}}}^{(2)}$	${{\mathcal {M}}}^{(3)}$	${{\mathscr {n}}}^{(1)}$	${{\mathscr {n}}}^{(2)}$	${{\mathscr {n}}}^{(3)}$	${{\mathscr {n}}}^{(4)}$	${{\mathscr {n}}}^{(5)}$
5	3	DN-121	RN-50	RN-18	(35,5)	(50,5)	(30,30)	(70,70)	(50,50)

‘RN’ stands for ResNet^23^ and ‘DN’ stands for DenseNet^24^ neural network architectures, respectively.

Synthetic errors (incorrect labels) were added to the training dataset of 18,991 images, which is split 80/20 ($k=5$) into training and validation sets and where for each study *t*, ${\varvec{\mathscr {n}}}^{(t)}=\left( {\varvec{\mathscr {n}}}_{A}^{(t)},{\varvec{\mathscr {n}}}_{B}^{(t)},{\varvec{\mathscr {n}}}_{M}^{(t)},{\varvec{\mathscr {n}}}_{T}^{(t)}\right)$ represents fractional levels of incorrect labels for airplane (*A*), boat (*B*), motorcycle (*M*), and truck (*T*) classes (see Table 5), respectively, where $0\le {\varvec{\mathscr {n}}}(\%) \le 100$, and where R stands for ResNet^23^, and D stands for DenseNet^24^. Note, in Table 5, the fractional level of incorrect labels was kept constant across classes in each study, so only one value is shown.

**Table 5:** Parameters used to test untrainable data cleansing (algorithm 1) for a multi-classification problem.

*k*	*n*	${\mathcal {{M}}}^{(1)}$	${\mathcal {{M}}}^{(2)}$	${\mathcal {{M}}}^{(3)}$	${\mathcal {{M}}}^{(4)}$	${\mathscr {{n}}}^{(0)}$	${\mathscr {{n}}}^{(1)}$	${\mathscr {{n}}}^{(2)}$	${\mathscr {{n}}}^{(3)}$	${\mathscr {{n}}}^{(4)}$	${\mathscr {{n}}}^{(5)}$	${\mathscr {{n}}}^{(6)}$	${\mathscr {{n}}}^{(7)}$	${\mathscr {{n}}}^{(8)}$	${\mathscr {{n}}}^{(9)}$
5	4	D-121	R-101	R-50	R-18	0	10	20	30	40	50	60	70	80	90

### Reader consensus between radiologists for correct vs. noisy labels

Images identified by UDC to have noisy labels are suspected to have inconsistencies rendering their annotation (or labeling) more difficult. As such, we expect the reader consensus of Pneumonia/Normal assessments between different radiologists to be lower for images with noisy labels than for those with correct labels that are easily identified by the AI model and for which we expect a relatively high reader consensus between radiologists. The following two hypotheses are formulated and can be directly tested using the (Cohen’s) kappa test:

- ${\varvec{\mathcal {H}}}_\mathbf{0} ^\mathbf {(1)}$: The level of agreement between radiologists for noisy labels is different from random chance.
- ${\varvec{\mathcal {H}}}_\mathbf{a} ^\mathbf {(1)}$: The level of agreement between radiologists for noisy labels is no different from random chance.
- ${\varvec{\mathcal {H}}}_\mathbf{0} ^\mathbf {(2)}$: The level of agreement between radiologists for correct labels is no greater than random chance.
- ${\varvec{\mathcal {H}}}_\mathbf{a} ^\mathbf {(2)}$: The level of agreement between radiologists for correct labels is greater than random chance.

We prepare an experimental dataset by splitting the data into correct and noisy labels as follows, where the two subsets are used in a clinical study to test the above hypotheses and validate UDC:

1. A dataset $\mathcal {D}$ with 200 elements ${\varvec{\mathscr {z}}}_j = \left( \mathbf {x}_j,\hat{y}_j\right)$ has images $\mathbf {x}_j$ and (noisy) annotated labels $\hat{y}_j$. This dataset is split into two equal subsets of 100 images each:
  - $\mathcal {D}_{clean}$—labels identified as correct by UDC, with the following breakdown:
    i. 48 Normal
    ii. 52 Pneumonia (39 Bacterial / 13 Viral)
  - $\mathcal {D}_{noisy}$—labels identified as noisy by UDC, with the following breakdown:
    i. 51 Normal
    ii. 49 Pneumonia (14 Bacterial / 35 Viral)
2. The dataset $\mathcal {D}$ is randomized to create a new dataset $\hat{\mathcal {D}}$ for an expert radiologist to label, and to indicate confidence or certainty in those labels (Low, Medium, High). This randomization is to address fatigue bias and any bias related to the ordering of the images.
3. The *reader consensus* between the expert radiologist and the original labels is calculated using Cohen’s kappa test26 and is compared between datasets $\mathcal {D}_{clean}$ vs. $\mathcal {D}_{noisy}$.

Figure 1 provides visual evidence showing that both null hypotheses, ${\varvec{\mathcal {H}}}_\mathbf{0} ^\mathbf {(1)}$ and ${\varvec{\mathcal {H}}_0^{(2)}}$, are rejected with very high confidence ($>99.9\%$) and effect size ($>0.85$). Therefore, both alternate hypotheses are accepted: ${\varvec{\mathcal {H}}}_\mathbf{a} ^\mathbf {(1)}$, stating that labels identified as noisy have levels of agreement no different from random chance, and ${\varvec{\mathcal {H}}}_\mathbf{a} ^\mathbf {(2)}$, stating that labels identified by UDC as correct have levels of agreement greater than random chance.

## Supplementary Information 1

Supplementary Information 1.

Supplementary Information 2.
