# Supplementary figures and images for: Automated detection of poor-quality data: case studies in healthcare
Source: Sci Rep. 2021 Sep 9;11:18005. doi: 10.1038/s41598-021-97341-0 (PMC8429593; doi:10.1038/s41598-021-97341-0)

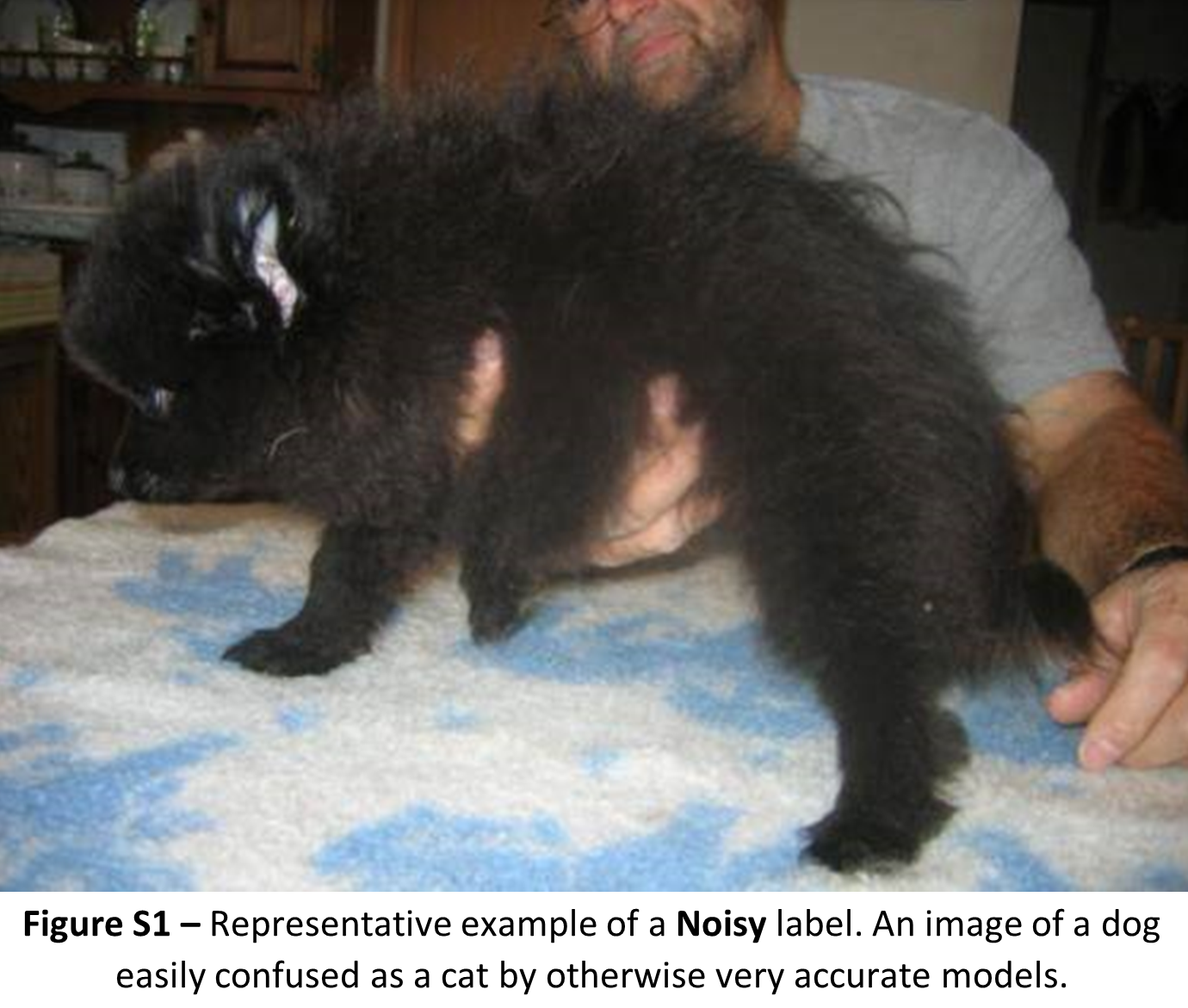

Supplement: Supplementary file 1 — Supplementary Information 1. [file 41598_2021_97341_MOESM1_ESM.png]

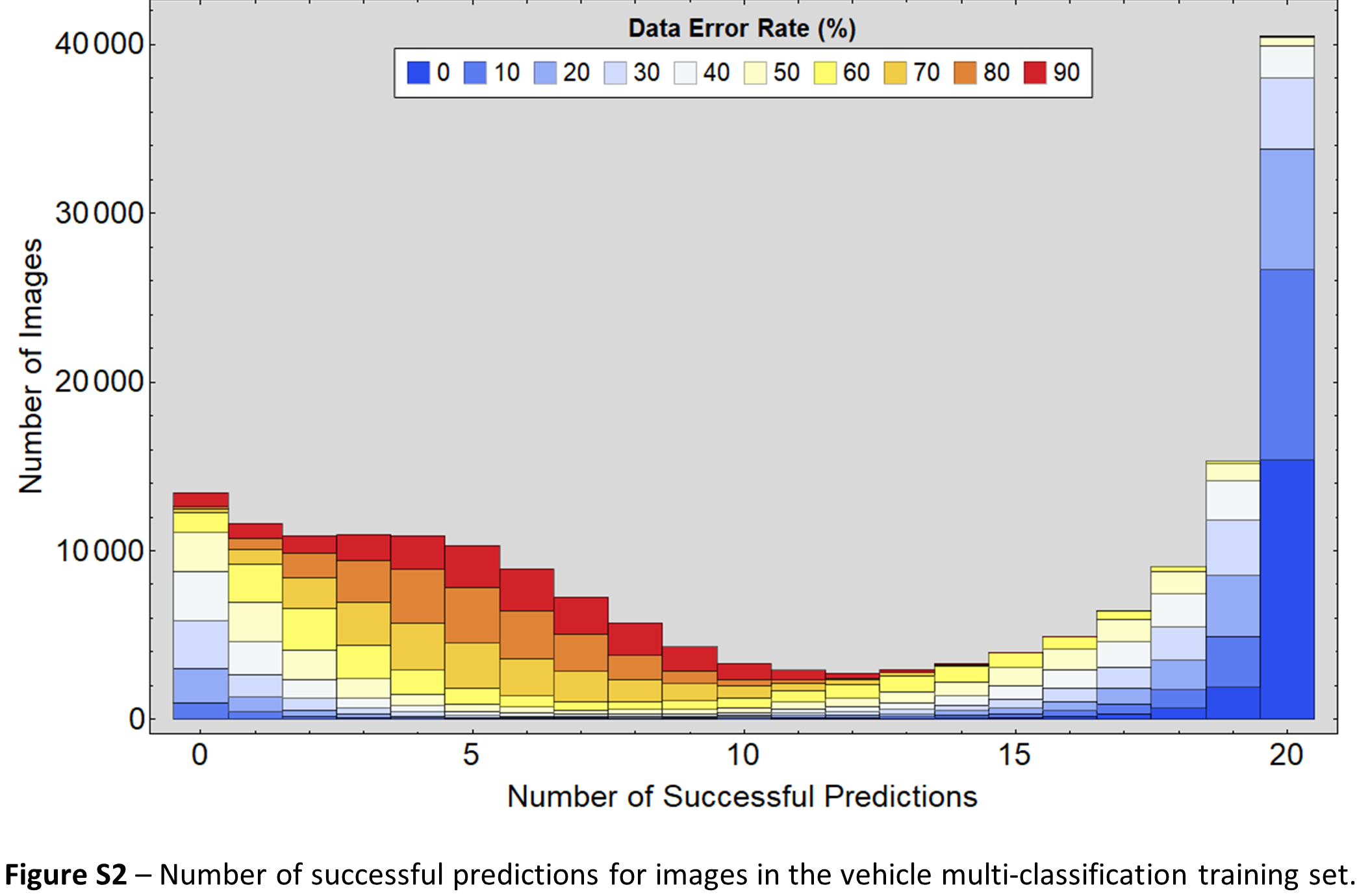

Supplement: Supplementary file 2 — Supplementary Information 2. [file 41598_2021_97341_MOESM2_ESM.png]
